# Plasma from patients with bacterial sepsis or severe COVID-19 induces suppressive myeloid cell production from hematopoietic progenitors in vitro

**DOI:** 10.1126/scitranslmed.abe9599

**Published:** 2021-06-16

**Authors:** Miguel Reyes, Michael R. Filbin, Roby P. Bhattacharyya, Abraham Sonny, Arnav Mehta, Kianna Billman, Kyle R. Kays, Mayra Pinilla-Vera, Maura E. Benson, Lisa A. Cosimi, Deborah T. Hung, Bruce D. Levy, Alexandra-Chloe Villani, Moshe Sade-Feldman, Rebecca M. Baron, Marcia B. Goldberg, Paul C. Blainey, Nir Hacohen

**Affiliations:** 1 Broad Institute of MIT and Harvard, Cambridge, MA, USA.; 2 Department of Biological Engineering, Massachusetts Institute of Technology, Cambridge, MA, USA.; 3 Department of Emergency Medicine, Massachusetts General Hospital and Harvard Medical School, Boston, MA, USA.; 4 Center for Bacterial Pathogenesis, Division of Infectious Diseases, Department of Medicine, Massachusetts General Hospital and Harvard Medical School, Boston, MA, USA.; 5 Department of Anesthesia, Critical Care, and Pain Medicine, Massachusetts General Hospital and Harvard Medical School, Boston, MA, USA.; 6 Center for Cancer Research, Massachusetts General Hospital and Harvard Medical School, Boston, MA, USA.; 7 Division of Pulmonary and Critical Care Medicine, Department of Medicine, Brigham and Women’s Hospital and Harvard Medical School, Boston, MA, USA.; 8 Center for Immunology and Inflammatory Diseases, Massachusetts General Hospital and Harvard Medical School, Boston, MA, USA.; 9 Koch Institute for Integrative Cancer Research at MIT, Cambridge, MA, USA.

## Abstract

Bacterial sepsis and severe COVID-19 share similar clinical manifestations and are both associated with dysregulation of the myeloid cell compartment. We previously reported an expanded CD14^+^ monocyte state, MS1, in patients with bacterial sepsis and validated expansion of this cell subpopulation in publicly available transcriptomics data. Here, using published datasets, we show that the gene expression program associated with MS1 correlated with sepsis severity and was up-regulated in monocytes from patients with severe COVID-19. To examine the ontogeny and function of MS1 cells, we developed a cellular model for inducing CD14^+^ MS1 monocytes from healthy bone marrow hematopoietic stem and progenitor cells (HSPCs). We found that plasma from patients with bacterial sepsis or COVID-19 induced myelopoiesis in HSPCs in vitro and expression of the MS1 gene program in monocytes and neutrophils that differentiated from these HSPCs. Furthermore, we found that plasma concentrations of IL-6, and to a lesser extent IL-10, correlated with increased myeloid cell output from HSPCs in vitro and enhanced expression of the MS1 gene program. We validated the requirement for these two cytokines to induce the MS1 gene program through CRISPR-Cas9 editing of their receptors in HSPCs. Using this cellular model system, we demonstrated that induced MS1 cells were broadly immunosuppressive and showed decreased responsiveness to stimulation with a synthetic RNA analog. Our in vitro study suggests a potential role for systemic cytokines in inducing myelopoiesis during severe bacterial or SARS-CoV-2 infection.

## Myeloid suppressive cells enter the arena

Patients with bacterial sepsis or severe COVID-19 have increased numbers of suppressive myeloid cells in their blood. Reyes *et al*. now show that similar cells can be induced by treatment of healthy human bone marrow progenitor cells in culture with plasma from patients with bacterial sepsis or severe COVID-19. The differentiation of suppressive myeloid cells in this cell model depended on the cytokines IL-6 and IL-10, indicating a role for systemic factors in inducing myeloid dysregulation in patients with severe infections.

## Teaser

Cytokines in plasma from sepsis or severe COVID-19 patients induce production of suppressive myeloid cells from hematopoietic progenitors in vitro.

## INTRODUCTION

A recent estimate suggests that one in five deaths globally is associated with sepsis (*1*). To date, no targeted treatment is available for this syndrome, likely because of substantial disease heterogeneity (*2*, *3*) and our lack of insight into sepsis immunopathology (*4*). These issues are highlighted by the current coronavirus disease 2019 (COVID-19) pandemic, wherein many clinical manifestations of severe acute respiratory syndrome coronavirus 2 (SARS-CoV-2) infection parallel those of bacterial sepsis (*5*–*8*). Sepsis is associated with profound alterations in the peripheral immune cell compartment, including a marked reduction in lymphocyte counts (*9*–*11*) and phenotypic alteration of myeloid cells (*12*–*14*). Monocytes from patients with sepsis have decreased responsiveness to stimuli (*14*–*16*) and have lower expression of human leukocyte antigen–DR (HLA-DR) (*17*–*22*), which is characteristic of monocytic myeloid-derived suppressor cells (MDSCs). In line with these findings, we recently reported an expanded CD14^+^ monocyte state in patients with sepsis called MS1, which is reminiscent of MDSCs (*23*). Previous studies performed in mice have reported opposing effects of MDSCs on sepsis outcomes (*24*–*27*), warranting further investigation into their ontogeny and function in humans and association with prognosis in patients with sepsis.

Recent reports have also noted the expansion of abnormal myeloid cells in the blood of patients with severe COVID-19 (*7*, *28*–*31*). In particular, monocytes from patients with severe SARS-CoV-2 infection show increased expression of calprotectin (*S100A8* and *S100A9*) and EN-RAGE (*S100A12*) and reduced expression of class II major histocompatibility complex (MHC-II) (*7*, *28*, *30*). Furthermore, myeloid cells from severe cases of COVID-19 have a reduced interferon (IFN) response, which is associated with diminished capacity for viral control (*7*, *29*). These studies provide valuable data describing the phenotypic alteration of myeloid cells in severe COVID-19; however, they do not provide insight into the specific mechanisms through which these abnormal myeloid cells are produced. Identifying specific factors that result in the induction of these cells will facilitate the study of these cells in greater detail and further our understanding of their function during SARS-CoV-2 infection.

Here, we identified the gene expression programs associated with the MS1 cell state and correlated these programs with disease severity in patients with bacterial sepsis or COVID-19. We showed that the MS1 cell state was induced in CD34^+^ human hematopoietic stem and progenitor cells (HSPCs) in vitro after incubation with plasma from patients with bacterial sepsis or severe COVID-19. We generated MS1 monocytes and neutrophils from HSPCs in vitro and demonstrated that they were immunosuppressive; we then showed that the cytokines interleukin-6 (IL-6) and IL-10 contributed to MS1 gene program induction.

## RESULTS

### Expression of the MS1 and MHC-II gene program is associated with sepsis severity

**Fig. 1:**
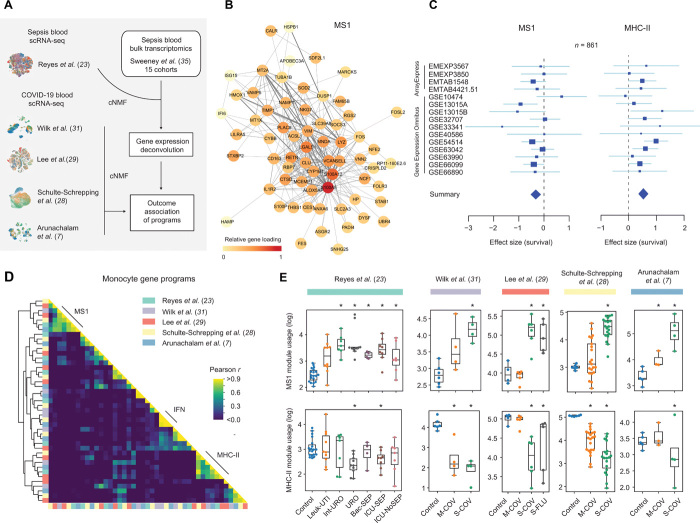
The MS1 gene expression program is associated with disease severity in bacterial sepsis and COVID-19. (**A**) Shown is the analysis scheme for 5 scRNA-seq datasets and 15 bulk transcriptomics datasets from cohorts of patients with bacterial sepsis or COVID-19. (**B**) Shown is a correlation network for the MS1 gene expression program in monocytes from patients with bacterial sepsis. Edge thickness is proportional to the correlation value between each pair of genes. Node colors are proportional to the expression level in log(transcripts per million) of each gene in the program. (**C**) Forest plots indicate the effect size on patient survival (log_2_ standardized mean difference) of inferred MS1 (left) or MHC-II (right) gene expression program usage in each dataset (patient cohort) from bulk gene expression deconvolution. Accession numbers for the data from each dataset are listed on the left. Blue boxes indicate the effect size in an individual study, with whiskers extending to the 95% confidence interval. Size of the box is proportional to the relative sample size of the study. Blue diamonds represent the summary effect size among the patient groups, determined by integrating the standardized mean differences across all studies. The width of the diamond corresponds to its 95% confidence interval. (**D**) Correlation matrix of the gene weights (*z* scores) for the monocyte gene expression programs across five scRNA-seq datasets for cohorts of patients with bacterial sepsis or COVID-19 is presented (table S7) (*7*, *23*, *28*, *29*, *31*). Gene expression modules were derived in an unbiased manner from each dataset using consensus non-negative matrix factorization (cNMF). (**E**) Mean usage (log) of the MS1 (top) and MHC-II (bottom) gene expression programs in monocytes from each patient across patient groups for each dataset (cohort) is shown. Asterisks indicate a false discovery rate (FDR) < 0.05, computed by comparing each disease state with that of healthy controls (two-tailed Wilcoxon rank sum test, corrected for testing of multiple modules). Boxes show the median and interquartile range (IQR) for each patient cohort, with whiskers extending to 1.5 IQR in either direction from the top or bottom quartile. Detailed descriptions of the patient cohorts and numbers of cells and patients for each of the five datasets in (D) and (E) are described in the corresponding publications (*7*, *23*, *28*, *29*, *31*) and in table S7. Control, healthy controls; Leuk-UTI, urinary tract infection with leukocytosis; Int-URO, intermediate urosepsis; URO, urosepsis; Bac-SEP, sepsis with confirmed bacteremia; ICU-SEP, intensive care with sepsis; ICU-NoSEP, intensive care without sepsis; M-COV, mild COVID-19; S-COV, severe COVID-19; S-FLU, severe influenza A.

To characterize the gene expression programs associated with the MS1 cell state, we analyzed monocyte single-cell RNA sequencing (scRNA-seq) datasets from four cohorts including patients with sepsis and healthy controls from our earlier study (*23*) using consensus non-negative matrix factorization (cNMF; Fig. 1A and fig. S1, A to D) (*32*). We found a gene expression program that included the MS1 marker genes *RETN*, *ALOX5AP*, and *IL1R2* among the top 20 genes with highest loadings (fig. S1D). This gene program was expressed more highly in patients with sepsis compared to healthy controls [false discovery rate (FDR) < 0.001; fig. S1E], and its expression correlated with the fractional abundance of MS1 cells in each patient (*r* = 0.73, *P* < 0.001; fig. S1F). Expression of the MS1 gene program negatively correlated with expression of the MHC-II program (fig. S1G), which was consistent with our observation that MS1 cells have lower surface expression of HLA-DR (*23*). Coexpression analysis within the MS1 program revealed that most genes were associated with *S100A8* (Fig. 1B), suggesting a role for *S100A8* as a driver of the MS1 gene expression program. This gene and its partner *S100A9* have been implicated in the development of MDSCs in cancer (*33*) and sepsis (*24*, *34*), indicating a similarity between monocytic MDSCs and the MS1 cell state.

We hypothesized that expression of the MS1 gene program may be associated with worse outcomes in bacterial sepsis. In transcriptional data from our earlier study (*23*), expression of the MS1 gene program correlated with sepsis severity in patients in the intensive care unit (ICU) but not in patients presenting to the hospital emergency department with milder disease (fig. S1H). Using gene expression deconvolution, we estimated the usage of the monocyte gene expression programs in 15 cohorts included in a recent meta-analysis examining sepsis mortality (fig. S1C and table S1) (*35*). We found that expression of the MS1 gene program was negatively associated with patient survival (effect size = −0.32, FDR < 0.01), whereas expression of the MHC-II gene program had the opposite relationship (effect size = 0.53, FDR < 0.01; Fig. 1C).

### Similar MS1 and MHC-II gene expression programs are observed in severe COVID-19

Gene expression signatures similar to that of MS1 have also been described recently in severe SARS-CoV-2 infection (*28*, *36*) but have not been systematically analyzed across multiple cohorts. To determine whether MS1 cells are similarly expanded in severe COVID-19, we analyzed four independent COVID-19 scRNA-seq datasets (*7*, *28*, *29*, *31*) independently and identified gene expression programs in CD14^+^ monocytes from each dataset using an unbiased cNMF method (Fig. 1A and fig. S2). In each of the four COVID-19 datasets, we found gene expression programs corresponding to the MS1 and MHC-II modules, as evidenced by their strong correlation (Pearson *r* > 0.8) with gene expression programs from our sepsis datasets (Fig. 1D). Similar to the trends that we observed in bacterial sepsis, CD14^+^ monocytes from patients with severe SARS-CoV-2 infection or influenza A infection had higher and lower usage of the MS1 and MHC-II gene programs, respectively (FDR < 0.05; Fig. 1E), and showed increased MS1 scores compared to healthy controls (*P* < 0.01; fig. S2). These findings suggest that the MS1 cell state is expanded in both bacterial sepsis and severe viral infection syndromes.

### Treating HSPCs with sepsis or severe COVID-19 plasma induces the MS1 gene program

**Fig. 2:**
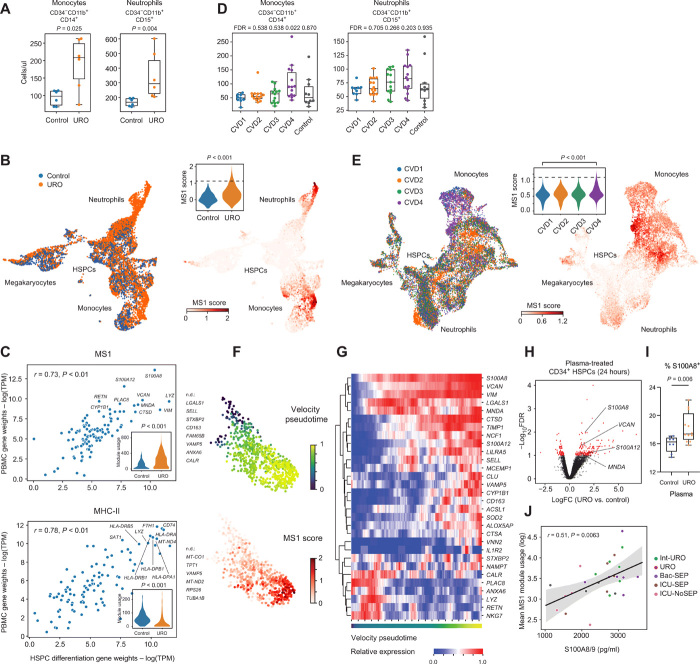
Sepsis and COVID-19 plasma samples induce myeloid differentiation of HSPCs and MS1 gene program expression in monocytes. (**A**) Shown is the number of CD34^−^CD11b^+^CD14^+^ (left) and CD34^−^CD11b^+^CD15^+^ (right) myeloid cells produced after incubation of CD34^+^ HSPCs in vitro with control plasma or plasma from patients with urosepsis for 7 days. Six experiments were performed for each condition in (A) (three plasma donors with two technical replicates). *P* values were calculated using a two-tailed Wilcoxon rank sum test. (**B**) Shown are uniform manifold approximation and projection (UMAP) projections of scRNA-seq data from the experiment with HSPCs incubated with urosepsis plasma shown in (A). Colors indicate the plasma pool with which the CD34^+^ HSPCs were treated (left) or the MS1 gene expression score for each cell (right). Major immune cell types are labeled on the basis of expression of known marker genes. The experiment in (B) was performed on CD34^+^ HSPCs from two healthy bone marrow donors with two plasma donors for each condition; a total of 3039 and 5254 cells were profiled for the control plasma and urosepsis plasma treatment, respectively. (**C**) Gene weight correlations between the MS1 gene expression program (top) or MHC-II gene expression program (bottom) in experiments with HSPCs incubated with urosepsis plasma (*x* axis) and in PBMCs from patients with sepsis (*y* axis) are shown. Significance of the correlations (Pearson *r*) was calculated with a permutation test. Genes that were not detected (n.d.) in the HSPC-plasma incubation experiment but were among the top 30 genes for the corresponding gene program in the PBMC dataset are indicated. Insets show violin plots of gene program usage across different plasma treatment conditions. TPM, transcripts per million. (**D**) Shown is the number of CD34^−^CD11b^+^CD14^+^ (left) and CD34^−^CD11b^+^CD15^+^ (right) myeloid cells produced after 7 days of incubation of CD34^+^ HSPCs with plasma from patients that were not infected (Control, *n* = 10 patients) or infected with SARS-CoV-2 [CVD1 (*n* = 9), non-hospitalized; CVD2 (*n* = 14), hospitalized; CVD3 (*n* = 14), ICU; CVD4 (*n* = 10), deceased]. FDR values are shown when comparing plasma for each disease state to mild COVID-19 (CVD1) patient plasma (two-tailed Wilcoxon rank sum test, corrected for testing of multiple cohorts). (**E**) Shown are UMAP projections of scRNA-seq data from experiments incubating CD34^+^ HSPCs with COVID-19 plasma. Colors indicate the plasma pool with which the CD34^+^ HSPCs were treated (left) or the MS1 gene expression score for each cell (right). Major immune cell types are labeled on the basis of expression of known marker genes. The experiment in (E) was performed with HSPCs from two healthy bone marrow donors using pooled plasma from all donors in (D); a total of 4449, 4591, 3129, and 3711 cells were profiled after incubation of HSPCs with plasma from patients with mild to severe COVID-19, respectively. Inset shows violin plots of MS1 gene expression scores for CD14-expressing cells from each plasma treatment condition. Dashed line indicates the mean MS1 score in cells from the MS1 cluster in the PBMC dataset (*23*). (**F**) Shown is a UMAP projection of the MS1 cell cluster differentiating from CD34^+^ HSPCs in vitro. Cells are colored by relative RNA velocity pseudotime (top) (*70*), which is derived from the ratio of spliced and unspliced transcripts in the scRNA-seq dataset, and MS1 score (bottom). (**G**) A clustered heatmap of the top 30 genes in the MS1 module is presented. Columns indicate individual cells ordered by velocity pseudotime. Expression values are *z* score normalized for each gene. (**H**) Volcano plot shows differential gene expression analysis (exact test) between CD34^+^ HSPCs treated with control or urosepsis plasma for 24 hours. Genes with FDR of <0.1 are highlighted in red, and selected genes that are up-regulated early in the MS1 gene expression program are labeled. Four experiments were performed for each condition (two plasma donors with two technical replicates). (**I**) Quantitation of S100A8 intracellular staining of CD34^+^ HSPCs treated with control or urosepsis plasma for 24 hours is shown. Four experiments were performed with six donors for control and urosepsis plasma, with two technical replicates for each plasma sample. (**J**) Shown is the correlation between S100A8/9 concentrations in plasma and MS1 gene expression for plasma samples from patients with urosepsis and controls (*n* = 28). Line and shadow indicate linear regression fit and 95% confidence interval, respectively. Significance of the correlation (Pearson *r*) was calculated with a two-sided permutation test.

We previously demonstrated that MS1-like cells could be derived from immature progenitor cells through stimulation of total bone marrow mononuclear cells (BMMCs) with lipopolysaccharide or Pam3CSK4 (*23*). Because BMMCs contain a heterogeneous mix of HSPCs and mature immune cells, potential paracrine interactions among the cell types confound identification of the precise factors that cause MS1 induction. Given this limitation, we sought a method for inducing the MS1 program directly in CD34^+^ HSPCs purified from healthy human bone marrow. We hypothesized that cytokines circulating in the blood of patients with sepsis might induce the differentiation of MS1 cells directly from HSPCs in vitro. Upon culturing HSPCs isolated from healthy human bone marrow with plasma from patients with urosepsis (urinary tract infection with organ dysfunction) or healthy controls, we found that sepsis plasma stimulated the production of monocytes and neutrophils more than healthy plasma did (*P* = 0.025 and 0.004 for CD34^−^CD11b^+^CD14^+^ and CD34^−^CD11b^+^CD15^+^ cells, respectively; Fig. 2A). Single-cell analysis of the differentiated cell populations showed clear trajectories of myeloid differentiation (Fig. 2B and fig. S3, A to D). We observed that incubation of HSPCs with plasma from patients with urosepsis resulted in the emergence of CD14^+^ cells with high MS1 scores compared with control plasma (*P* < 0.01; Fig. 2B). cNMF analysis of the scRNA-seq datasets generated in the plasma incubation experiments identified gene expression programs similar to the MS1 and MHC-II programs in patient peripheral blood mononuclear cells (PBMCs) (*23*), as evidenced by strong correlations between their gene loadings (Pearson *r* = 0.73 and 0.78, respectively; Fig. 2C and fig. S3C). The MS1 and MHC-II gene expression programs were also significantly up-regulated or down-regulated (*P* < 0.01), respectively, in CD14^+^ cells derived from HSPCs incubated with sepsis plasma in vitro (Fig. 2C). These data support our hypothesis that cytokines circulating in the blood of patients with sepsis could induce the differentiation of MS1 cells from HSPCs in vitro.

We next hypothesized that similar effects might be observed when incubating HSPCs in vitro with plasma from patients with severe COVID-19. We performed the same experiments with heat-inactivated plasma from SARS-CoV-2–infected patients with mild to severe disease (CVD1-CVD4) and uninfected controls (fig. S3, E and F, and table S2). Plasma from COVID-19 patients who eventually died (CVD4) stimulated the production of monocytes more strongly than did plasma from nonhospitalized patients (CVD1) (FDR = 0.02; Fig. 2D), although to a weaker extent than did plasma from patients with sepsis. Compared with plasma from CVD1 patients, plasma from CVD4 patients induced the production of CD14^+^ cells with higher MS1 scores (FDR < 0.01; Fig. 2E) and caused increased and decreased expression of the MS1 and MHC-II gene programs, respectively (*P* < 0.01; fig. S3, G and H). These findings suggested that circulating factors in the blood of patients with severe COVID-19 could stimulate the induction of MS1 cells from HSPCs in vitro.

### An MS1-like gene program is expressed in neutrophils from the blood of sepsis patients and HSPCs treated with sepsis plasma

Upon incubation of HSPCs with urosepsis plasma, we observed cells with high MS1 scores and a gene expression program similar to that of neutrophils produced from differentiating HSPCs (Fig. 2B and fig. S3C). Neutrophils from the blood of critically ill patients with sepsis and patients with bacteremia expressed a gene program that included *S100A8* among genes with the highest loadings (fig. S4, A to C) and correlated with the MS1-like module in neutrophils generated from HSPCs treated with urosepsis plasma (Pearson *r* = 0.58; fig. S4D). MS1 marker genes were also among the top differentially expressed genes in neutrophils from critically ill patients with sepsis or bacteremic patients compared to healthy individuals (fig. S4E). These findings are consistent with previous reports of MDSCs having both granulocytic and monocytic subtypes (*37*) and highlight the similarity between the MS1 cell state and MDSCs.

### Up-regulation of S100A8 is associated with expression of the MS1 gene program

Analysis of monocyte differentiation from HSPCs in vitro showed different pseudo-temporal dynamics of MS1 gene expression (Fig. 2F). A number of genes including *S100A8*, *VCAN*, and *MNDA* were expressed during early differentiation and remained up-regulated (Fig. 2G). Short-term stimulation (24 hours) of CD34^+^ HSPCs with sepsis plasma resulted in the up-regulation of these genes (Fig. 2H and table S3) and an increase in the fraction of S100A8^+^ cells in the CD34^+^ population (Fig. 2I). These genes were among the core genes expressed in the MS1 program (Fig. 1B), and their early up-regulation was in line with our hypothesis that *S100A8* was an important factor driving the induction and differentiation of MS1 cells from HSPCs. In addition, the plasma concentrations of the S100A8/9 dimer correlated significantly with the MS1 gene expression program in patients with sepsis (Pearson *r* = 0.51, *P* < 0.01; Fig. 2J).

### Induction of myelopoiesis in sepsis and COVID-19 depends on IL-6

**Fig. 3:**
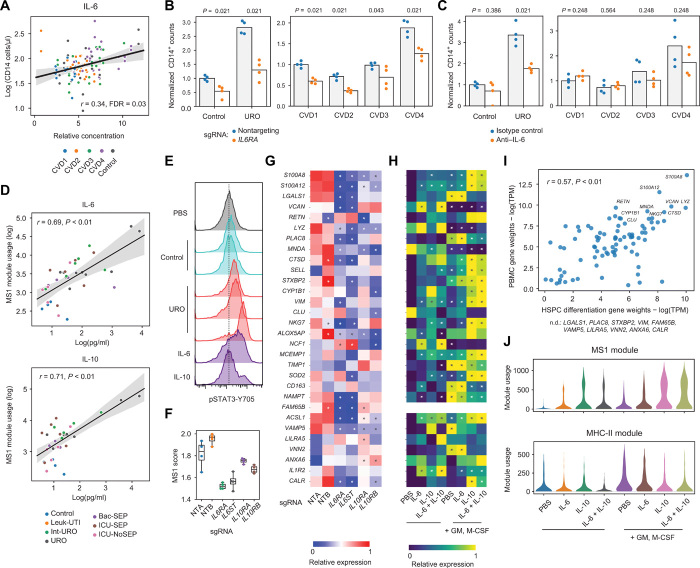
MS1 gene expression program induction in CD34^+^ HSPCs depends on IL-6 and IL-10. (**A**) Shown is the correlation between relative IL-6 concentrations in plasma from patients with COVID-19 and controls (normalized to total protein expression) and the production of CD34^−^, CD11b^+^, CD14^+^ monocytic cells after incubation of HSPCs with plasma from patients with COVID-19 (*n =* 51) or controls (*n* = 10). Line and shadow indicate linear regression fit and 95% confidence interval, respectively. Significance of the correlation (Pearson *r*) was calculated with a two-sided permutation test that was corrected for testing of multiple cytokines. (**B**) Shown is the CD34^−^, CD11b^+^, CD14^+^ monocytic cell production after incubation of CD34^+^ HSPCs in vitro with COVID-19 patient plasma (CVD1 to CVD4), urosepsis plasma, or control plasma for 7 days. HSPCs were electroporated with Cas9 ribonucleoproteins complexed with single guide RNAs (sgRNAs) targeting *IL6RA* or nontargeting (control). CD14^+^ cell counts are normalized to the mean of either the control (left) or mild COVID-19 (right) plasma condition for each bone marrow donor. (**C**) Shown is CD34^−^, CD11b^+^, CD14^+^ monocytic cell production after incubation of CD34^+^ HSPCs with COVID-19 plasma (CVD1 to CVD4), urosepsis plasma, or control plasma for 7 days in medium containing anti–IL-6 antibody or isotype antibody control. CD14^+^ cell counts are normalized to either the control (left) or mild COVID-19 (right) plasma condition for each bone marrow donor. Experiments in (B) and (C) were performed with HSPCs from two bone marrow donors with two technical replicates each, using pooled plasma from five independent patients or controls for each plasma condition. *P* values are calculated using a two-tailed Wilcoxon rank sum test. (**D**) Correlations between IL-6 (top) and IL-10 (bottom) concentrations in plasma and expression of the MS1 gene expression program are shown for 40 patients with sepsis and controls. Line and shadow indicate linear regression fit and 95% confidence interval, respectively. Significance of the correlations (Pearson *r*) was calculated with a two-sided permutation test. (**E**) Quantification of intracellular staining for phosphorylated STAT3 (Y705) in CD34^+^ HSPCs treated with control or urosepsis plasma or with IL-6 or IL-10 (100 ng/ml) is shown. Dashed line indicates median fluorescence for PBS-treated HSPCs. Results are representative of two independent experiments using different bone marrow donors. (**F**) MS1 gene expression scores were calculated from bulk RNA-seq of sorted CD14^+^ cells generated from CD34^+^ HSPCs electroporated with Cas9 ribonucleoproteins and treated with plasma from patients with urosepsis for 7 days. (**G**) Shown is expression of the top 30 MS1 genes in sorted CD14^+^ cells generated from CD34^+^ HSPCs electroporated with Cas9 ribonucleoproteins and treated with plasma from patients with urosepsis for 7 days. Asterisks indicate a significant difference (FDR < 0.1; Wilcoxon rank sum test, corrected for testing of multiple genes) compared with nontargeting guide RNA (NTA). In (F) and (G), *n* = 4 experiments were performed for each guide RNA condition (two biological and two technical replicates). (**H**) Expression of the top 30 MS1 genes from scRNA-seq data from CD14^+^ monocytic cells generated from CD34^+^ HSPCs treated with the indicated cytokines (all at 100 ng/ml) is shown. Asterisks indicate a significant difference (FDR < 0.1; Wilcoxon rank sum test, corrected for testing of multiple genes) compared with the PBS control. (**I**) Shown is the gene weight correlation between the MS1 gene expression program detected in the cytokine treatment (*x* axis) and patient PBMC datasets (*y* axis). Significance of the Pearson correlations (*r*) was calculated with a permutation test. Genes that were not detected in the cytokine treatment dataset but were among the top 30 genes in the PBMC dataset are indicated. (**J**) Violin plots show MS1 (top) and MHC-II (bottom) gene expression programs in CD14^+^ monocytic cells across the different cytokine treatments. The experiments in (H) to (J) were performed on HSPCs from two bone marrow donors for each cytokine treatment; a total of 3365, 2986, 2550, 3025, 3194, 3061, 2850, and 2918 cells for each cytokine treatment were profiled.

To determine which circulating factors in plasma induced the increased production of myeloid cells from HSPCs in vitro, we analyzed the concentrations of inflammatory cytokines implicated in cytokine storm (*38*) in plasma from patients with COVID-19 (fig. S5). We found that IL-6 concentrations correlated with a higher production of CD14^+^ cells from HSPCs in vitro (Fig. 3A), suggesting that this cytokine may be involved in the induction of myelopoiesis. To test this hypothesis, we added plasma from patients with urosepsis or COVID-19 or from corresponding healthy controls, to CD34^+^ HSPCs or CD34^+^ HSPCs lacking the IL-6 receptor and observed a marked reduction in CD14^+^ cell production in the latter group (Fig. 3B). We also observed a weaker reduction in CD14^+^ cell production from HSPCs in vitro specifically for urosepsis plasma when HSPCs were differentiated in the presence of IL-6 neutralizing antibodies (Fig. 3C). These findings suggested a role of circulating IL-6 in inducing myelopoiesis during severe infections.

### IL-6 and IL-10 induce MS1 program expression in monocytes differentiated from HSPCs

To determine the specific cytokines that induced the differentiation of MS1 cells from HSPCs in vitro, we measured the concentrations of inflammatory cytokines in the plasma of patients with sepsis and controls from our previous study (*23*). Several cytokines displayed increased concentrations in the plasma from patients with sepsis (fig. S6), but both IL-6 and IL-10 concentrations specifically correlated with the MS1 gene expression program in monocytes (Fig. 3D). This suggested the involvement of these two cytokines in MS1 cell induction and was consistent with their role in MDSC expansion in cancer (*39*, *40*). We found that short-term incubation of HSPCs with urosepsis plasma or IL-6, and variably with IL-10, but not with control plasma, resulted in phosphorylation of signal transducer and activator of transcription 3 (STAT3) (Fig. 3E), the transcription factor downstream of both IL-6 and IL-10 (*41*, *42*). In addition, short-term treatment of HSPCs in vitro with recombinant IL-6 or IL-10 showed a dose-dependent up-regulation of the early MS1 genes *S100A8*, *VCAN*, and *MNDA* (fig. S7A) and an increase in S100A8^+^ progenitor cells (fig. S7B), similar to the effects observed with sepsis plasma.

To further test these dependencies, we used CRISPR-Cas9 to knock out the surface receptors for IL-6 (*IL6RA* and *IL6ST*) and IL-10 (*IL10RA* and *IL10RB*) in CD34^+^ HSPCs before incubation with urosepsis plasma in vitro (fig. S7, C and D). Knockout of the IL-6 receptor resulted in the production of CD14^+^ cells with lower MS1 scores from HSPCs (Fig. 3F) and reduced expression of several, but not all, MS1 genes (Fig. 3G). In addition, in these experiments, the CD14^+^ cells displayed increased surface expression of HLA-DR, consistent with the absence of the MS1 phenotype (fig. S7E). Similar trends were observed with knockout of the IL-10 receptor albeit with weaker effects.

To test whether these two cytokines were sufficient to induce the MS1 gene expression program, we differentiated CD34^+^ HSPCs in the presence of IL-6 or IL-10 and with or without granulocyte-macrophage colony-stimulating factor (GM-CSF) and M-CSF, which are known growth factors that support the differentiation of HSPCs into monocytes (fig. S8) (*43*). We found that addition of GM-CSF and M-CSF, and to a lesser extent IL-6 or IL-10, increased the fraction of CD14^+^ cells produced by HSPCs, but IL-10 decreased the absolute number of cells produced. We found increased expression of several MS1 genes in CD14^+^ cells generated from HSPCs treated with IL-6 or IL-10 in the presence of GM-CSF and M-CSF (Fig. 3H). cNMF analysis revealed gene expression programs resembling MS1 and MHC-II programs, both of which correlated significantly with the gene expression programs of PBMCs from patients with sepsis (Pearson *r* = 0.57 and 0.66, *P* < 0.01; Fig. 3I). Expression of these programs showed opposite trends with IL-6 and IL-10 treatment (Fig. 3J), consistent with the negative correlation observed in the PBMC dataset (fig. S1G).

### The MS1 program in COVID-19 is associated with inflammatory macrophage activation

Previous studies suggested that increased activation of inflammatory macrophages in infection sites is associated with disease severity in both bacterial and SARS-CoV-2 infections (*44*–*46*). Because neither MS1 monocytes nor other peripheral immune cells express notable concentrations of IL-6 and IL-10 (*23*), we hypothesized that the increased concentrations of these cytokines in plasma were due to inflammatory activation of macrophages at sites of infection. We analyzed published scRNA-seq datasets from bronchoalveolar lavage fluid (BALF) samples and nasopharyngeal swabs from patients with SARS-CoV-2 infection and found significantly increased MS1 scores among monocytes and macrophages in patients with severe COVID-19 (*P* < 0.01; fig. S9, A and B). We also found that subsets of these cells expressed *IL6*, *IL10*, or multiple chemokines (*CCL3*, *CCL4*, *CXCL4*, and *CXCL8*) and cytokines (*TNF*, *IL1B*, and *IL1A*) corresponding to inflammatory activation of macrophages. We observed that the expression of both *IL6* and *IL10*, and the inflammatory macrophage gene program, positively correlated with expression of the MS1 program in each patient (fig. S9, C to H).

### MS1 monocytes suppress T cell proliferation and inflammatory activation of epithelial and endothelial cells

**Fig. 4:**
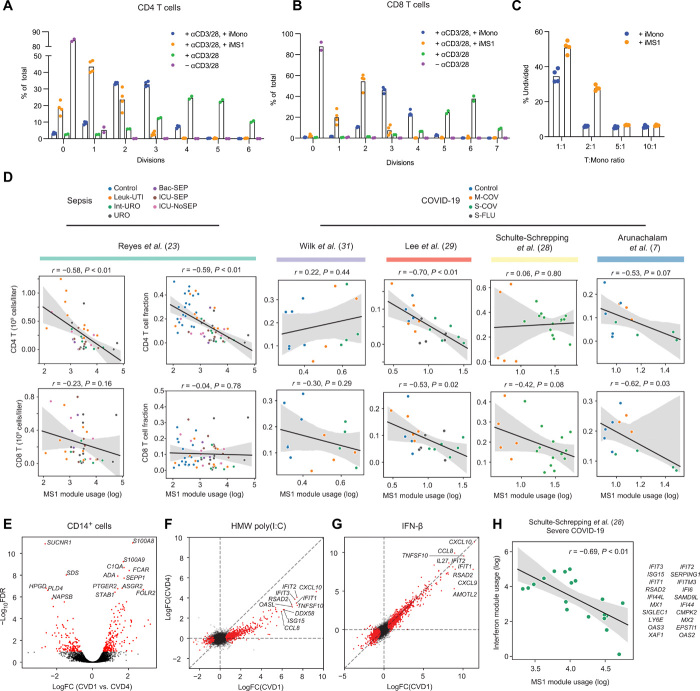
MS1 cells generated from HSPCs in vitro are immunosuppressive. (**A** and **B**) Shown is the number of divisions (after 4 days in culture) of (A) CD4 T cells and (B) CD8 T cells activated with anti-CD3 and anti-CD28 antibodies in vitro and incubated 1:1 with either iMono or iMS1 cells (induced by cytokines) generated from CD34^+^ HSPCs. Percentages are determined by CFSE (carboxyfluorescein diacetate succinimidyl ester) dilution and flow cytometry. (**C**) Fraction of nondividing CD4 T cells activated with anti-CD3 and anti-CD28 antibodies and co-incubated with either iMono or iMS1 cells at different ratios. In (A) to (C), four experiments were performed for each condition (two bone marrow donors, each with two technical replicates). (**D**) Scatterplots indicate the correlation between mean MS1 gene expression usage in monocytes and absolute abundance or fraction of (top) CD4 T cells or (bottom) CD8 T cells for each patient with bacterial sepsis (*23*) or COVID-19 (*7*, *28*, *29*, *31*) (each dot represents one patient). Line and shadow indicate linear regression fit and 95% confidence interval, respectively. Significance of the correlation (Pearson *r*) was calculated with a two-sided permutation test. (**E**) Volcano plot showing differential gene expression analysis results (exact test) between CD14^+^ cells generated from HSPCs in vitro in response to pooled plasma from patients with mild (CVD1) or severe (CVD4) COVID-19. Genes with FDR of <0.1 are in red and >0.1 are in black, and the top 15 genes with lowest FDR values are shown. (**F** and **G**) Scatterplots show the log_2_ fold change (FC) for each gene after high–molecular weight (HMW) poly-(I:C) (F) or IFN-β (G) treatment of CD14^+^ cells generated from HSPCs in vitro using pooled plasma from patients with mild (CVD1) COVID-19 on the *x* axis or severe COVID-19 (CVD4) on the *y* axis. Genes with FDR < 0.1 in CVD1 plasma-generated CD14^+^ cells are shown in red, and the top 10 genes with the highest fold change values are indicated. In (E) to (G), four experiments were performed for each condition (two bone marrow donors with two technical replicates). (**H**) Correlation between the IFN response and MS1 module usage in CD14^+^ monocytes from patients with severe COVID-19 is shown. Line and shadow indicate linear regression fit and 95% confidence interval, respectively. Significance of the correlation (Pearson *r*) was calculated with a two-sided permutation test. The top 20 genes associated with the IFN response are listed on the right.

Given their similarity to MDSCs, we hypothesized that MS1 cells may suppress the activation of T cells (*47*). We generated monocytes from CD34^+^ HSPCs either with GM-CSF and M-CSF alone (iMono) or with IL-6, IL-10, GM-CSF, and M-CSF (iMS1) and co-incubated the cells with allogeneic PBMCs activated with anti-CD3 and anti-CD28 antibodies. We found that coculture of T cells with iMS1 cells suppressed T cell proliferation, as evidenced by a reduction in the number of cell divisions undergone by both CD4 and CD8 T cells after 4 days of coculture (Fig. 4, A and B). The suppression of T cell proliferation by iMS1 also depended on the ratio of monocytes added to the coculture (Fig. 4C). Consistent with this finding, we found that expression of the MS1 gene module in monocytes correlated negatively with the fraction and absolute numbers of CD4 T cells, but not CD8 T cells, among total PBMCs from patients with sepsis compared to controls from our earlier study (Pearson *r* = −0.58 and −0.59, *P* < 0.01; Fig. 4D) (*23*). These results are consistent with previous studies showing that MDSCs in patients with sepsis have immunosuppressive effects on T cells (*21*, *48*). In published datasets comprising PBMC samples from patients with COVID-19 (*7*, *28*, *29*, *31*), we found that increased expression of the MS1 gene program in monocytes consistently correlated with lower CD8 T cell fractions in each of the four datasets that we analyzed (*P* < 0.05 in two of four datasets; Fig. 4D) and less so with CD4 T cells (*P* < 0.05 in one dataset) (*49*).

Sepsis is a systemic disease, so we tested the effects of co-incubating iMS1 cells with cell types from other organs that are commonly dysfunctional in sepsis (fig. S10A) (*50*). We found that co-incubation of iMS1 cells with tumor necrosis factor–α (TNFα)–activated human umbilical cord endothelial cells (HUVECs) resulted in lower expression of cytokines and chemokines (*CXCL2*, *CXCL8*, and *IL6*) and down-regulation of the TNFα signaling pathway compared with co-incubation with iMono cells (fig. S10, B and C, and table S4). iMS1 cells exerted a similar albeit weaker effect on human renal epithelial cells, as evidenced by the down-regulation of a number of TNFα signaling pathway genes (*NFKBIA*, *CCL2*, *TNIP2*, and *RELB*; fig. S10D and table S5). These effects were not observed in HUVECs or in human renal epithelial cells incubated with conditioned medium from iMS1 or iMono cells, suggesting a dependence on cell-cell contacts (fig. S10E). Co-incubation of human renal epithelial cells with iMS1 cells resulted in the up-regulation of *MMP1*, an important regulator of tissue remodeling and extracellular matrix homeostasis that has been previously shown to be up-regulated during the development of sepsis (*51*). Similarly, *PROS1*, the gene encoding protein S, an important regulator of the clotting cascade, was also up-regulated in human renal epithelial cells co-incubated with iMS1 cells, suggesting a possible involvement of MS1 cells in sepsis-related coagulopathy (*52*, *53*).

### Stimulated MS1 monocytes have a decreased IFN response

We next generated CD14^+^ cells from HSPCs in vitro using plasma from patients with mild (CVD1) or severe (CVD4) COVID-19. As expected, the core MS1 gene *S100A8* was the most significantly up-regulated gene in CD14^+^ cells generated from HSPCs using severe COVID-19 plasma (Fig. 4E and table S6). Next, we stimulated these CD14^+^ cell populations with high–molecular weight poly-(I:C), a synthetic RNA analog, or IFN-β, an important mediator of antiviral responses. We observed a weaker induction of various cytokines and IFN-stimulated genes in CD14^+^ cells generated with plasma from patients with severe COVID-19 in response to poly-(I:C), whereas no such effect was observed in response to IFN-β (Fig. 4, F and G). Consistent with these observations, we found that expression of the MS1 gene program in CD14^+^ cells correlated negatively with expression of IFN response genes in monocytes from patients with severe COVID-19 from a published study (*P* < 0.01; Fig. 4H) (*28*). These results correlate with our findings in bacterial sepsis (*23*), wherein MS1 cells showed a diminished response to pathogen-associated molecular patterns, and are consistent with a recent report demonstrating functional impairment of myeloid cells in patients with COVID-19 (*7*).

## DISCUSSION

Given the similarity between MS1 cells and monocytic MDSCs, we propose that the MS1 gene expression program could provide a precise definition for monocytic MDSCs in peripheral blood. Indeed, using MDSC data from a recent study (*54*), we found that ~50% of genes expressed in monocytic MDSCs, but not in monocytes, overlapped with MS1-specific genes (fig. S11). However, our single-cell-based MS1 gene signature contains many additional genes, and thus may more accurately define the MDSC subsets in sepsis. Our MS1 gene signature should be analyzed in MDSCs obtained from patients with other diseases (*47*, *55*–*57*). Given the multiple mechanisms underlying T cell suppression by MDSCs suggested in cancer studies (*58*–*60*), a systematic analysis of the interaction between myeloid cells and T cells in severe infections is needed to determine the exact mechanism behind the lymphopenia observed in patients with bacterial sepsis and severe COVID-19.

In this study, we show that the systemic circulating cytokines IL-6 and to a lesser extent IL-10 induce differentiation of HSPCs in culture into myeloid cells that express the MS1 gene expression program. Our findings provide a potential explanation for the expansion of suppressive myeloid cells in bacterial sepsis and severe COVID-19. Our study reveals that induction of myelopoiesis and the MS1 gene expression program in HSPCs in vitro depends on systemic IL-6, a current therapeutic candidate for treating severe COVID-19 (*61*). Our results suggest that the role of IL-6 in infection is complex and more extensive than just the induction of an acute phase immune response (*62*), consistent with the observed lack of an impact of IL-6 blockade in randomized clinical trials in patients with COVID-19 (*63*, *64*). Consistent with our experimental findings, a recent study has shown that treatment with tocilizumab, an anti-IL6-R antibody, resulted in decreased fractions of monocytic MDSCs in the blood of patients with severe COVID-19 (*65*).

Our analysis supports a model in which cytokine production by inflammatory macrophages at sites of infection may result in increased concentrations of systemic IL-6 and IL-10, promoting the expansion of myeloid cells expressing the MS1 gene program. We propose that the expansion and recruitment of immunosuppressive MS1 cells could serve as a negative feedback response to dampen excessive inflammation and tissue damage at infection sites, which in certain situations may be clinically beneficial. This expansion of immunosuppressive MS1 cells could perhaps be sustained by a feed-forward loop wherein an increase in systemic S100A8/9 protein released by MS1 cells could further stimulate Toll-like receptor 4 and lead to continued production of suppressive myeloid cells from progenitor cells (*23*, *66*).

We also demonstrate that MS1 cells generated from HSPCs in vitro have an anti-inflammatory effect on endothelial and epithelial cell lines, raising the possibility that MS1 cells exert their functional effects on nonimmune cell types during bacterial sepsis and severe COVID-19. This supports the hypothesis that the interaction of MS1 cells with other tissues affects pathogenesis of severe infections and suggests the need for profiling the function of myeloid cells from patients with severe infections and examining their impact on other cell types.

A key limitation of our study is the in vitro nature of the experiments. The role of cytokine production at sites of infection in stimulating HSPCs in the bone marrow must still be confirmed in animal models of sepsis and COVID-19. Furthermore, additional studies in vivo are needed to determine whether suppressive myeloid cells play a causal role in the development of or protection against organ dysfunction during sepsis and severe COVID-19.

## MATERIALS AND METHODS

### Study design

Plasma samples from patients with bacterial sepsis or healthy controls were obtained from an existing cohort of patients as described previously (*23*). COVID-19 plasma samples were obtained from patients entering the Massachusetts General Hospital (MGH) Emergency Department. This study was approved by the Partners Healthcare Institutional Review Board under protocol no. 2017P001681. Patients presenting to the MGH Emergency Department from March through May 2020 with respiratory distress suspected or known to be due to COVID-19 were enrolled. Inclusion criteria were age 18 years or older, clinical concern for COVID-19 upon Emergency Department presentation, and acute respiratory distress with at least one of the following: (i) tachypnea ≥ 22 breaths per minute, (ii) oxygen saturation ≤ 92% on room air, (iii) a requirement for supplemental oxygen, or (iv) a requirement for positive-pressure ventilation. Patients were categorized on the basis of disease outcomes as follows: CVD1, nonhospitalized (mild disease); CVD2, hospitalized without intensive care; CVD3, hospitalized and admitted to intensive care; and CVD4, hospitalized and eventually died; controls were SARS-CoV-2–negative patients. Blood samples were collected upon hospital presentation. None of the patients had a bloodstream infection, and only one patient in the CVD4 group had a possible coexisting bacterial infection (urinary tract). All of the patients with COVID-19 were adjudicated to have SARS-CoV-2 as a primary infection.

No prior sample size calculations were performed. Sample size was determined to be adequate on the basis of the degree and consistency of differences between groups. The number of cells, biological replicates, and technical replicates are indicated in each figure legend. Investigators were not blinded during experiments.

Patient cohorts, sample numbers, and sample types for the transcriptomic datasets analyzed in this study are detailed in table S7. Briefly, we analyzed seven scRNA-seq datasets, including those from PBMCs, BALF, and nasopharyngeal swab samples from patients with bacterial sepsis or COVID-19. To determine the association of monocyte gene programs with sepsis mortality, we analyzed a compilation of bulk transcriptomic datasets from a previous meta-analysis (*35*).

### Bone marrow CD34^+^ progenitor cell isolation and culture

Purified CD34^+^ bone marrow cells from healthy individuals were either purchased directly from STEMCELL Technologies or isolated from fresh human bone marrow. Bone marrow aspirates anticoagulated with EDTA were purchased from StemExpress and processed within 24 hours of isolation. To remove red blood cells (RBCs) from the bone marrow, 1× RBC lysis buffer (eBioscience) was added directly at a 10:1 ratio to the sample. After 5 min, the cells were centrifuged at 400*g* for 5 min and resuspended in 1X RBC lysis buffer to further clear the sample of RBCs. The cells were then centrifuged, resuspended in fluorescence-activated cell sorting (FACS) buffer [1X phosphate-buffered saline (PBS), 2.5% fetal bovine serum, and 2 mM EDTA; Invitrogen] and purified using human CD34 MicroBeads (Miltenyi Biotec). Isolated CD34^+^ cells were validated using flow cytometry (CD34-BV650, clone 561, BioLegend) to be of >90% purity. Cells were cryopreserved in CryoStor CS10 (StemCell Technologies) in aliquots of 200,000 cells each. The tubes were kept at −80°C overnight and then transferred to liquid nitrogen for long-term storage.

For each experiment, CD34^+^ cells were first thawed and rested for 48 hours in SFEM II (StemCell Technologies) with 75 nM StemRegenin 1 (StemCell Technologies), 3.5 nM UM171 (StemCell Technologies), stem cell factor (SCF), thrombopoietin (TPO), and FMS-related receptor tyrosine kinase 3 ligand (Flt3L) (40 ng/ml) (PeproTech), and 1X penicillin-streptomycin (Gibco). Cells were subsequently cultured in the same medium supplemented with either 20% plasma sterilized through a 0.2-μm filter (Millipore) or 100 ng/ml of the cytokines IL-6, IL-10, GM-CSF, and M-CSF (PeproTech). iMS1 and iMono cells were isolated after 7 days of culture using human CD14 MicroBeads (Miltenyi Biotec). Isolated CD14^+^ cells were validated using flow cytometry [CD14-FITC (fluorescein isothiocyanate), clone M5E2, BioLegend] to be of >90% purity.

### Flow cytometry for assessing myeloid output

To assess the number of myeloid cells from plasma incubation experiments, cells were stained with the following panel: CD3-APC (allophycocyanin) (clone HIT3a), CD19-APC (clone HIB19), CD56-APC (clone 5.1H11), CD14-FITC (clone M5E2), CD15-AF700 (clone HI98), CD11b-PE-Cy7 (clone ICRF44), CD34-BV650 (clone 561), and CD38-PE (phycoerythrin)/Cy5 (clone HIT2) (BioLegend). After staining, cells were resuspended in FACS buffer with 2% CountBright beads (Invitrogen) to allow determination of absolute counts during analysis. Flow cytometry data were acquired on a CytoFLEX LX (Beckman Coulter) and analyzed using FlowJo v10.1.

### scRNA-seq and data analysis

scRNA-seq combined with cell hashing (*67*) was performed as previously described (*23*). Briefly, cells from multiple culture conditions were labeled with hashtag oligo (HTO) antibodies (BioLegend) and loaded on the Chromium platform using the 3′ v3 profiling chemistry (10X Genomics). Libraries were sequenced to a depth of ~25,000 reads per cell on a NextSeq 550 (Illumina). The data were aligned to the GRCh38 reference genome using cellranger v3.1 (10X Genomics). Because of their technical incompatibility with droplet-based platforms, scRNA-seq of neutrophils was performed using plate-based Smart-Seq2 as previously described (*68*).

Single-cell data analysis was performed using scanpy (*69*) with the same preprocessing and filtering parameters described in a prior publication (*23*). To identify the major cell types in the differentiation experiments, we assessed the expression of the following marker genes: HSPCs, *CD34* and *CD38*; monocytes, *CD14* and *LYZ*; neutrophils, *ELANE* and *MPO*; and megakaryocytes, *PPBP* and *PF4*. MS1 scores were calculated for the top 30 genes from the MS1 module derived from the sepsis dataset using the “score_genes” in scanpy (ctrl_size = 50, n_bins = 25). RNA velocity analysis was performed using the scVelo package (*70*) using the default parameters.

cNMF analysis was performed as detailed in a previous publication (*32*). Briefly, the top 3000 variable genes for each dataset were first selected to filter the gene expression matrix. NMF was then performed with *k* = 5 to 25 (10 iterations for each *k*). The number of modules (*k*) for downstream analysis was selected on the basis of biological interpretability of the modules and stability of the cNMF solution. To ensure that no modules from technical artifacts were analyzed, only gene programs with mean usage of >10 across all cells were included for further analysis.

### Bulk data deconvolution and meta-analysis

The gene loading matrix from cNMF analysis of monocytes was used to construct a reference matrix for gene expression deconvolution. To reduce the number of genes in the reference matrix, only the top 1000 variable genes within the monocyte data were included. Sepsis datasets with survival annotation were obtained from a previously published meta-analysis (*35*). Gene expression deconvolution was performed using CIBERSORT (*71*) with a no-sum-to-one constraint and absolute scoring. The resulting score matrix was then used as an input to MetaIntegrator (*72*), where the effect size of each gene module was visualized using forest plots.

### Plasma collection

Plasma samples from patients with bacterial sepsis were isolated by obtaining the top layer from Ficoll gradient separation of whole blood (diluted 1:1 with 1X PBS) and were centrifuged again at 1000*g* for 10 min to remove cell debris. Samples were immediately stored at −80°C. Before use in experiments, plasma samples were quickly thawed at 37°C. Plasma samples from patients with bacterial sepsis were not subjected to heat inactivation.

For patients with COVID-19, plasma samples were obtained as described above, but using whole blood diluted 1:2 in RPMI 1640. Before use in experiments, plasma samples were quickly thawed at 37°C and incubated for 1 hour at 53°C to inactivate viral particles. For the knockout and scRNA-seq experiments, plasma samples were pooled across each patient category.

### Blood samples for neutrophil sorting and scRNA-seq

Samples for neutrophil scRNA-seq were from patients enrolled in the Brigham and Women’s Hospital (BWH); the criteria for patient recruitment for this cohort are described elsewhere (*73*, *74*). Control samples consisted of blood samples from age, gender, and ethnicity-matched healthy controls obtained from Research Blood Components (MA, USA).

To maintain viability of neutrophils for scRNA-seq, fresh blood was collected in EDTA Vacutainer tubes (BD Biosciences) and processed within 4 hours. To remove RBCs from the sample, 1X RBC lysis buffer (eBioscience) was added directly at a 10:1 ratio to the sample. After 5 min, the cells were centrifuged at 400*g* for 5 min and resuspended in 1X RBC lysis buffer to further clear the sample of RBCs. The remaining cells were stained with a general panel: 4′,6-diamidino-2-phenylindole, CD3-APC (clone HIT3a), CD19-APC (clone HIB19), CD56-APC (clone 5.1H11), CD14-FITC (clone M5E2), CD15-AF700 (clone HI98), and CD11b-PE-Cy7 (clone ICRF44) (BioLegend). Single neutrophils were sorted using an SH800 cell sorter (Sony) into 10 μl of TCL buffer (Qiagen) with 1% β-mercaptoethanol (BME; Sigma-Aldrich) in individual wells of a 96-well plate.

### Bulk RNA-seq processing and data analysis

Bulk RNA-seq was performed using Smart-Seq2 (*75*) with minor modifications, as described previously (*76*), using 1000 cells as input. All RNA-seq libraries were sequenced with 38 × 38 paired-end reads using NextSeq (Illumina). RNA-seq libraries were sequenced to a depth of >2 million reads per sample. STAR was used to align sequencing reads to the UCSC hg19 transcriptome, and RSEM was used to generate an expression matrix for all samples. Both raw count and transcripts per million data were analyzed using edgeR and custom python scripts.

### Intracellular protein staining

Intracellular staining with S100A8-PE (clone REA917, Miltenyi Biotec) was performed using the Cytofix/Cytoperm kit (BD Biosciences) following the manufacturer’s protocol. For staining of phosphorylated STAT3 (Y705), cells were first fixed with 4% paraformaldehyde (Electron Microscopy Sciences) for 15 min at room temperature. The cells were then washed twice with 1X PBS and resuspended in 95% ice-cold methanol and left at −20°C overnight. The permeabilized cells were then stained with a pSTAT3-Y705 antibody (clone 13A3-1, BioLegend) for 30 min on ice. Flow cytometry data were acquired on a CytoFLEX LX (Beckman Coulter) and analyzed using FlowJo v10.1.

### CRISPR-Cas9 editing of CD34^+^ HSPCs

Cas9 protein, predesigned guide RNAs targeting *S100A8*, *S100A9*, *IL6ST*, *IL6R*, *IL10RA*, and *IL10RB*, and nontargeting guide RNAs (from GeCKO v2 library) were purchased from Integrated DNA Technologies. Ribonucleoprotein complexes were assembled by combining 2.1 μl of 1X PBS, 1.2 μl of 100 μM guide RNA, and 1.7 μl of Cas9 protein (10 μg/ml) and incubating at room temperature for 15 min. The complexes were added to 50,000 to 100,000 CD34^+^ HSPCs resuspended in 20 μl of P3 (Lonza) and electroporated (program code DZ-100) using the 4D-Nucleofector system (Lonza). After electroporation, the cells were immediately transferred to 500 μl of HSPC media and rested for 48 hours. Knockout efficiency in CD34^+^ HSPCs was assessed after 48 hours via flow cytometry using the following panel: CD34-BV650 (clone 561), CD38-PE/Cy5 (clone HIT2), CD126-APC (clone UV4), and CD210-PECy7 (clone 3F9) (BioLegend).

### Plasma protein measurements

Samples from patients with sepsis and controls were thawed and analyzed in parallel using the LEGENDplex Human Inflammation for IFN-α2, IFN-γ, IL-1β, IL-6, IL-10, TNFα, and IL-18; the LEGENDplex Human Hematopoietic Stem Cell Panels for M-CSF and GM-CSF; and the LEGENDplex Human Vascular Inflammation Panel for S100A8/9 (BioLegend). Flow cytometry data were acquired on a CytoFLEX LX (Beckman Coulter) and analyzed using FlowJo v10.1.

Samples from patients with COVID-19 and controls were analyzed using a commercially available multiplexed proximity extension assay (Olink Proteomics) as detailed by Filbin *et al.* (*77*). Normalized protein expression (NPX) values are calculated from the number of mapped counts on a NovaSeq run (Illumina) for each protein and represent a relative quantification scale (log_2_ fold change over the mean of all proteins across all plasma samples).

### T cell coculture and proliferation assay

T cell coculture was performed as previously described (*78*) with minor modifications. Briefly, tissue culture plates were coated with purified anti-CD3 (5 μg/ml) (clone HIT3a, BioLegend) at 4°C overnight and subsequently washed twice with 1X PBS. PBMCs from a healthy donor (Research Blood Components) were labeled with carboxyfluorescein diacetate succinimidyl ester (CFSE) (Invitrogen) following the manufacturer’s protocol. PBMCs were resuspended in SFEM II (StemCell Technologies) with purified anti-CD28 (5 μg/ml) (clone CD28.2, BioLegend) and plated at a density of 1 million cells/ml. Isolated iMS1 or iMono cells were added at different ratios as indicated. The cells were left in culture for 3 to 4 days, with media replenished after 2 days. At the end of incubation, the cells were stained with CD3-AF700 (clone OKT3), CD4-APC (clone OKT4), and CD8a-PE (clone RPA-T8) (BioLegend) to determine the amount of CFSE dilution within the T cells.

### HUVEC and human renal epithelial cell coculture and flow sorting

Primary HUVECs and human renal epithelial cells were purchased from Lonza and cultured in EGM-2 and REGM, respectively. To improve cell viability, HUVECs were cultured in tissue culture plates precoated with Matrigel (Corning) diluted 1:100 in EBM-2 (Lonza). Cells were used for experiments within three to five passages. Before coculture, HUVECs and human renal epithelial cells (HREs) were treated with TNFα (10 ng/ml) (PeproTech) for 24 hours. For the coculture experiments, CFSE-labeled iMono or iMS1 cells were added at a 1:1 ratio to a confluent monolayer of HUVECs or HREs and incubated for 2 hours. The cells were washed three times with 1X PBS and detached by adding 1X Accutase (Innovative Cell Technologies). The cells were transferred to FACS buffer after 15 min, and 1000 CFSE-negative cells were sorted into 10 μl of TCL buffer (Qiagen) with 1% BME (Sigma) for bulk RNA-seq. Conditioned media were prepared by incubating iMS1 or iMono cells at 0.5 M cells/ml in EGM-2 or REGM for 24 hours overnight.

### Stimulation of CD14^+^ cells generated from HSPCs treated with COVID-19 plasma

CVD1 and CVD4 plasma-generated CD14^+^ cells were isolated after 7 days of culture using human CD14 MicroBeads (Miltenyi Biotec). Isolated CD14^+^ cells were validated using flow cytometry (CD14-FITC, clone M5E2; BioLegend) to be of >90% purity. Cells were stimulated for 4 hours with IFN-β (100 ng/ml) (PeproTech) or high–molecular weight poly-(I:C) (1 μg/ml) (InvivoGen). After stimulation, cells were washed twice with FACS buffer and resuspended in 20 μl of TCL buffer (Qiagen) with 1% BME (Sigma) for bulk RNA-seq.

### Statistical analysis

Statistical analyses were performed with either GraphPad Prism 8 software or the SciPy statistics package. For most analyses, *P* values were calculated using two-tailed Wilcoxon rank sum tests and corrected for multiple testing using the Benjamini-Hochberg method. Differential gene expression analyses for bulk RNA-seq data were performed using edgeR exact tests.

## SUPPLEMENTARY MATERIALS

stm.sciencemag.org/cgi/content/full/scitranslmed.abe9599/DC1
Figs. S1 to S11 Tables S1 to S7 Data files S1 to S4

## Appendix

View/request a protocol for this paper from *Bio-protocol*.
